# Book Notices

**Published:** 1858-02

**Authors:** 


### BOOK NOTICES.

PRIZE ESSAY: RATIONAL THERAPEUTICS, or the Comparative Value of Different Curative Means, and the Principles of their Application. "*Natura Duce.*" By WORTHINGTON HOOKER, M.D., of New Haven. 1857.

This is the essay to which the prize of one hundred dollars was awarded by the Massachusetts Medical Society, at its annual meeting, June 3, 1857. It is headed by the following sentence, extracted from the speech of Dr. A. A. Gould before that body, at its annual meeting in 1855: "We would regard every approach toward the rational and successful prevention or management of disease without the necessity of drugs to be an advance in favor of humanity and scientific medicine."

Dr. Hooker is well known to the profession as one of the most talented votaries of the science of medicine in this country. His pen, although not so prolific as some, is always wielded with signal ability. This essay is written in a spirit of temperate philosophy and earnest impartiality, and convinces the reader that the author is a man actuated by conscientious motives. The style is lucid and vigorous, while the matter contained in it evinces profound knowledge of the subject and a thorough acquaintance with the literature of the profession. Let everybody read it who is fond of *multum in parvo.*

We extract the following instructive observations on the use of mercury, as an example of his manner of dealing with the subject. The extract commences on page 22.

The change which has been made in the use of mercury, during the past twenty-five or thirty years, presents many points of interest. Its use for some purposes has been entirely discontinued. It was quite largely used for some length of time in fever, with the idea, that, if the constitution could be brought under its influence, the fever would be removed; a mercurial fever, as it might be called, taking its place, which the recuperative powers of nature could easily remove. Although in some cases this mode of practice seemed to produce the effect intended, it was found, on the whole, so frequently to fail, and to be attended sometimes with such disastrous results, that it has been wholly abandoned. Mercury is used, at the present time, very sparingly in fever, and only so far as it is needed to affect the secretions, or to combat accompanying inflammation.

Mercury is also discontinued from use in the exanthematous diseases, unless there be some special reasons in the complications of these diseases for its employment. At one time, its use was highly lauded by Dr. Armstrong and others as a remedy in scarlatina; but, at the present time, there is no truth more definitely settled by the experience of the profession, than the impropriety of its use as a common remedy in this malady.

Mercury is no longer used as a common cathartic. It has come to be the settled practice of the profession to avoid its use, in this respect, in all cases where the distinctive effects of this drug are not called for. This is in strong contrast with the incautious and indiscriminate use of this remedy which was so prevalent, both in and out of the profession, during the first quarter of this century.

The same discriminating experience which has discarded mercury as a general remedy in fever and in the exanthemata has retained it in the treatment of inflammations; but it has been found that it need not be pushed to the extent that was formerly supposed to be necessary in this class of diseases. In most cases, it is not necessary even to affect the gums; and salivation is always to be avoided; although, in some severe cases, it is proper to run the risk of producing it.

The combination of calomel, antimony, and opium, which, in various proportions, is now so much used, is a remedy of very great value in the treatment of inflammatory diseases. To Dr. Robert Hamilton, of Lynn Regis, England, the credit is commonly given of first drawing the attention of the profession, in the year 1783, to the efficacy of this combination. He says that its usefulness was first suggested to him in following out a hint given to him by an army surgeon, in relation to the use of calomel in the treatment of acute hepatitis. He added opium to the calomel to relieve the pain attendant upon inflammation, and then antimony to relieve the febrile excitement and to produce perspiration; and, inferring that this combination might be serviceable in the treatment of the inflammation of other organs as well as that of the liver, he proceeded to verify the inference, and thus inaugurated a practice which has become established by abundant experience as one of the permanent advances of the profession in the treatment of a very wide range of disease.

Mercury is a remedy of great value in the treatment of many chronic diseases; but, during the reign of active medication, it was used in them too largely, and often with so little discrimination, that disastrous results were produced. Not only is it now used much more cautiously in these maladies, but, in very many cases in which it would formerly have been deemed applicable, it is now given up, as calculated only to add to the sufferings of the patient, without effecting any good result, or to leave him in a bad condition after the cure of the particular disease for which it is used is accomplished, or even to prevent a cure which might have been effected if more gentle means had been employed. In Dr. James Hamilton's book on the "Use and Abuse of Mercurial Medicines," there are many interesting facts stated bearing upon these points.

Emphatically may it be said, in view of the sad results which have come from the needless use of mercury, that the great diminution of its use in the treatment of disease is "an advance in favor of humanity and scientific medicine." At the same time, it is to be borne in mind, that it is far from being a mere general diminution. It is a diminution which is based upon an extensive range of discriminations; so that, while in some cases where it was formerly used it is now wholly discarded, and in others it is used with much less freedom, there are some cases in which its introduction into the system is effected as rapidly as possible; and cases occasionally occur in which there is some reason to think that it is proper to use it in exceedingly large doses. This last point, however, is as yet *sub judice.*

Some of the most valuable acquisitions which the profession has made in therapeutics during the present century are the discriminating limitations that it has been able to put upon the use of this remedy, which is one of the most efficient of its active means of cure. The advance which has been effected in this respect step by step in the profession's experience is greater than is ordinarily supposed. Larger additions have in this way been made to our real means of cure than by any, or even perhaps all, of the new remedies that have been discovered during the same period of time.

All disturbing remedies are much less in vogue now than they were in the first quarter of this century. Physicians then very commonly used such remedies, especially in the beginning of attacks of disease, for the purpose of breaking up the attack, or of lessening its force. Emetics were common remedies for this object. The practice was applicable in some cases; but it was far too generally employed. So common was the plan of thus adding to the turmoil of disease at the outset, that it was a popular saying, that it was necessary to make one worse in order to make him better. And this disturbing and depressing mode of treatment was by no means confined to the beginning of disease; but it was customary to continue it to some degree during the progress of the case. Febrile symptoms, so long as they lasted, were combated actively; and active remedies were always addressed to the removal of any local derangements that might exist, little dependence being placed upon the recuperative efforts of nature. But now the general character of medical practice is vastly different in the points alluded to. In comparatively few cases, even of acute disease, is the patient made worse at the beginning, in order to make him better. Generally he is made better at once, by measures that relieve the disturbance of disease, instead of adding to it; and, during the progress of the case, great caution is exercised in the use of any remedies that may interfere with the rest and quiet so essential to the free operation of the recuperative powers, or that may so depress them that they cannot act with sufficient energy to effect a recovery. The truth that the irritation of disease is often the great source of the exhaustion attending it, and that the physician should therefore be careful not to add to it by his remedies, is now quite fully appreciated. Commonly, it is true, some disturbing treatment is required occasionally during the progress of a case; but it is managed with caution, and generally quieting remedies are used in combination, so as to render the disturbance as light as possible.

The change of which I have been speaking has done for medicine what the introduction of the art of healing by the first intention has done for surgery. The irritation of the disturbing modes of treatment so prevalent during the reign of active medication had the same effect upon internal maladies that the irritating ointments had upon the wounds into which they were inserted so universally by surgeons before the time of Ambrose Pare; and the improvement in both cases consists in a return to the simplicity of nature.

Perhaps there is no remedy in the use of which there has been so much change as bleeding. It was during the first quarter of this century, and even for some years farther on, a common remedy in all febrile and inflammatory diseases. I have already alluded to the views of Dr. Gallup in regard to this remedy. Although there were few in this country whose views were as extreme as his were, bleeding was everywhere a favorite remedy with the profession. In England, Armstrong and Southwood Smith were the prominent advocates of bleeding. The strong views of the latter—so earnestly, skilfully, and I may say beautifully, developed in his book on fever—were very captivating to all enthusiastic minds; and his book had for a time a wide influence, both in England and in this country.

Bleeding was popular with the people as well as with the profession. In almost all cases of accident, it was practised as a matter of course. In pregnancy, it was resorted to as the established remedy for various inconveniences and complaints, that we now find are easily removed by less formidable means, or that commonly had better be borne than be removed, if bleeding be the only thing that can remove them. It was the custom also with many to be bled occasionally, in order to guard against attacks of disease to which they supposed themselves liable. This was practised especially in the spring.

This very common resort to bleeding, both as a remedy and as a preventive, is now abandoned. This remedy has, with others, been subjected to discriminating limitations; perhaps, from the influence in part of popular prejudice, it has been in some quarters too much given up, especially local bleeding. Whenever inflammation exists in an important organ in any marked degree of severity, this remedy is applicable,—general bleeding when there is sufficient constitutional affection to call for it, and the system is in a condition to bear the loss of blood; and local bleeding when the circumstances of the case do not warrant general bleeding.

Bleeding, it is to be remembered, is a remedy that is calculated to allay the irritation of disease; and it never adds to it when it is really applicable, and is not made use of to an improper extent. It is therefore, in some cases, really not as objectionable as certain remedies that are substituted for it in order to avoid exhaustion. It is often better to reduce febrile excitement or inflammation by this quiet remedy than to do it with remedies that may exhaust the vital energies by a series of impressions which are depressing, and at the same time irritating. I am persuaded that there is often too little fear of this result from such impressions on the part of those who have great fear that bleeding would produce it. Remedies of the kind alluded to, when pushed too far, may cause an exhaustion as irremediable as that which is produced by an inappropriate bleeding.

